# Lipedema: Progress, Challenges, and the Road Ahead

**DOI:** 10.1111/obr.13953

**Published:** 2025-05-27

**Authors:** Vincenza Cifarelli

**Affiliations:** ^1^ Department of Pharmacology and Physiology Saint Louis University School of Medicine St. Louis Missouri USA

**Keywords:** adipose tissue, inflammation, lipedema, lymphatics, VEGF‐C

## Abstract

**Introduction:**

Lipedema is a chronic and progressive disease that predominantly affects women, characterized by a disproportionate increase in subcutaneous adipose tissue (AT), particularly in the lower limbs. It is associated with significant physical disability, chronic pain, thromboembolism, and psychosocial distress. Despite its profound impact on women's health and quality of life, lipedema remains underrecognized and insufficiently studied, with an estimated prevalence of approximately 10% among women worldwide. Although the exact etiology of lipedema remains unclear, emerging evidence suggests a multifactorial origin involving genetic predisposition, hormonal influences, and vascular dysfunction—all contributing to its development and progression. Current therapeutic options provide only partial symptom relief and remain noncurative, highlighting the urgent need for expanded research and improved management strategies.

**Methods:**

A systematic review was conducted to assess the current understanding of lipedema pathophysiology and current treatment options. Research articles were sourced from PubMed, Web of Science, ScienceDirect, and Scopus databases. Over 100 studies were incorporated.

**Results:**

This review provides a comprehensive overview of lipedema, encompassing its clinical features, pathophysiological mechanisms, diagnostic challenges, and current treatment modalities. Additionally, the review discusses whether the molecular and metabolic differences between abdominal and femoral AT depots mirror those observed in classical obesity.

**Conclusions:**

Multidisciplinary, research‐informed care is essential for managing lipedema, combining conservative therapies, tailored exercise, and liposuction for advanced cases. More research to better understand the underlying pathophysiology is critical to developing targeted treatments, improving diagnostic accuracy, and informing standardized, evidence‐based care.

## 1 Introduction

Lipedema is a chronic and progressive disease characterized by the disproportionate painful accumulation of subcutaneous adipose tissue (AT) in the hips, buttocks, and legs [1]. Lipedema occurs almost exclusively in women and usually develops during early adulthood due to stress, surgery, and/or hormonal changes [2]. Lipedema in males is rare, and it is usually associated with higher estrogen and lower testosterone levels [3] and severe liver disease [4]. Lipedema is associated with impaired mobility, venous thromboembolism (VTE) [5, 6], osteoarthritis, and chronic pain [7]. Despite the clinical impact on women's health and quality of life, lipedema is largely understudied and misdiagnosed as classical obesity, delaying appropriate treatment and worsening disease morbidity.

Indirect estimates suggest that lipedema may be more common than previously recognized. Epidemiological data, derived from the misdiagnosis of lipedema as lymphedema, indicate that the condition could affect 6%–11% of women in the general population [8, 9]. However, the true prevalence of lipedema remains unknown reflecting several barriers to accurate estimates. These include the following: (i) Although the World Health Organization classified lipedema as a distinct disease in the International Classification of Diseases (ICD‐11), the United States still uses ICD‐10 and cannot yet adopt the new code, with no clear timeline for transition. (ii) Awareness is lacking among healthcare providers who are unfamiliar with the condition and fail to identify cases in the clinical practice resulting in misdiagnosis with obesity or lymphedema, because of its association with increased AT deposition, or swelling. (iii) A definitive diagnostic test or biomarker for lipedema is absent, making the diagnosis reliant on clinical evaluation and patient history. This subjectivity contributes to the variability in the recognition and report of lipedema, further complicating efforts to measure its prevalence.

### 1.1 Lipedema Etiology

The etiology of lipedema remains unclear, though evidence points to a combination of genetic, hormonal, and vascular factors that contribute to its development and progression [10]. Although no single causative gene has been confirmed, familial clustering and inheritance patterns support a hereditary component, with variations in gene expression potentially influencing disease severity and progression. Family history is frequently reported, with many cases showing inheritance patterns that suggest a possible autosomal dominant mode with incomplete penetrance [11]. A familial case of lipedema has been linked to a mutation in the *AKR1C1* gene, encoding for an aldo‐keto reductase that catalyzes the reduction of progesterone to its inactive form, 20‐alpha‐hydroxyprogesterone [12]. In a different study, a mutation in the *PIT1* gene [3] (POU class 1 homeobox 1) was identified. This gene encodes for a transcription factor that regulates the expression of growth hormone prolactin and thyroid‐stimulating hormone beta, all of which are essential for the function of the endocrine system. However, sporadic cases complicate the genetic interpretation, suggesting the involvement of environmental factors or epigenetic mechanisms. Recent advances in genetic and transcriptomic analyses have begun to shed light on potential molecular pathways involved in lipedema. Michelini et al. [13], have recently proposed a genetic testing panel using next‐generation sequencing to identify potential genetic variations associated with the development of lipedema. The study conducted genomic sequencing on 162 Italian and American patients with lipedema, identifying 305 genes potentially associated with the condition and overlapping diseases relevant to lipedema. The analysis identified 21 deleterious variants across 12 genes (*PLIN1*, *LIPE*, *ALDH18A1*, *PPARG*, *GHR*, *INSR*, *RYR1*, *NPC1*, *POMC*, *NR0B2*, *GCKR*, and *PPARA*) in 17 patients [13]. More recently, a genome‐wide association study using an inferred lipedema phenotype identified 18 genetic risk factors in adult women of European descent using the UK Biobank [14]. Among those, loci located in or near *RSPO3*, *GRB14‐COBLL1*, *ZNF664‐FAM101A* (proximal to *CCDC92*), *VEGFA*, *ADAMTS9*, *LYPLAL1*, and *ANKRD55‐MAP 3K1* have been previously associated with waist‐to‐hip ratio [15, 16, 17]. Notably, these loci exhibit stronger effects in women compared to men, highlighting potential sex‐specific genetic influences on body fat distribution. Of note, two of these loci, namely, *VEGFA* and *GRB14‐COBLL1*, were significantly associated with lipedema in an independent case–control study that included clinically diagnosed lipedema cases. Vascular endothelial growth factor (VEGF)–A is an established regulator of angiogenesis and vessel remodeling. VEGF‐A‐mediated vascularization of AT is essential for maintaining metabolic homeostasis by ensuring proper oxygenation, nutrient supply, and waste removal [18]. A well‐developed and functional vascular network supports healthy AT expansion, preventing the development of hypoxia—a condition characterized by low oxygen levels—which can trigger inflammation, fibrosis, and metabolic dysfunction [19]. *VEGF* genetic variants, along with other 62 different loci, were recently reported to be significantly associated with both higher adiposity and lower cardiometabolic risk [20], in line with key clinical features of lipedema [21]. The growth factor receptor‐bound protein 14 (GRB14)/cordon‐bleu WH2 repeat protein‐like 1 (COBLL1) *locus* has been linked to body fat distribution [15, 22]. Although the precise molecular mechanisms are not completely understood, GRB14 and COBLL1 play distinct roles in adipocyte biology and insulin signaling. GRB14 inhibits insulin receptor signaling [23] by preventing the phosphorylation of downstream signaling molecules such as protein kinase B (PKB/AKT) and extracellular signal‐regulated kinase (ERK) [24, 25]. In murine primary hepatocytes, knockdown of Grb14 leads to a significant decrease in insulin‐induced processing and expression of sterol regulatory element‐binding protein 1c, highlighting its role in lipid metabolism [26]. In human preadipocytes, GRB14 knockout results in decreased differentiation efficiency, proliferation rate, and lipid storage. Conversely, COBLL1 knockout leads to excessive lipid storage and lipolysis without affecting adipogenesis or insulin‐stimulated AKT2 phosphorylation [27]. Despite these efforts, the genetic driver(s) of lipedema remains elusive, highlighting the need for follow‐up studies using larger and more diverse populations to further investigate the identified loci and associated genes, validate findings, and uncover additional loci. In addition, experimental studies to determine how these loci influence biological processes related to AT biology and lipedema‐specific clinical characteristics are also needed.

Lipedema predominantly affects women, often manifesting or worsening during hormonal transitions such as puberty, pregnancy, or menopause, implicating estrogen and progesterone in its progression [28]. In an “*Opinion Article*,” the concept of lipedema as a phenotypic manifestation of a “pseudopregnancy” was recently proposed [29]. In this model, lipedema could be considered a pathological condition where hormonal and physiological processes, similar to pregnancy, lead to abnormal fat accumulation and tissue remodeling, but without actual pregnancy occurring. Authors proposed that lipedema could be triggered by a selective accumulation of bacterial lipopolysaccharides (LPS), or endotoxin, in gluteofemoral white AT. During pregnancy, intestinal permeability increases as part of the body's adaptive responses to support the fetus. This condition, known as “leaky gut syndrome,” allows for an increased entry of bacterial endotoxins into circulation, leading to systemic low‐grade inflammation. In lipedema, this enhanced permeability might be a contributing factor in the accumulation of LPS in AT, particularly in the gluteofemoral region. This could exacerbate local inflammatory responses and contribute to fat accumulation and vascular dysfunction typical of lipedema. This model offers a different perspective on the disease's origins, underscoring the importance of understanding how systemic factors, such as gut health and the microbiota, might be driving local tissue remodeling in lipedema. Investigating these pathways could reveal new therapeutic avenues for addressing the root causes of this complex disease.

Microvascular and lymphatic abnormalities have been also proposed to be responsible for lipedema development and progression. Microangiopathy refers to small blood vessel abnormalities, including structural and functional impairments. In lipedema, this is hypothesized to manifest as capillary fragility, increased permeability, and dysregulated angiogenesis. Elevated VEGF plasma levels, as reported by Siems et al., suggest a role in promoting aberrant angiogenesis [30]. VEGF‐related changes may contribute to the development of abnormal vasculature and increased capillary permeability, exacerbating tissue hypoxia and inflammation. Increased capillary numbers and dilation have been reported in lipedema fat [31], suggesting defective angiogenesis independent of obesity. Dysfunctional venoarterial reflexes, often seen in lipedema patients, may further impair microvascular function, promoting edema and local inflammation [2]. However, existing studies are limited by small sample sizes, observational designs, and variability in histological criteria. In addition, similar vascular changes can occur in obesity complicating differentiation. Functional studies assessing capillary permeability, angiogenesis, and endothelial cell behavior in lipedema tissue are needed. In addition, longitudinal analysis would help determine whether microangiopathy precedes or is secondary to other pathophysiological changes typical of the disease.

This review aims to provide a comprehensive and up‐to‐date overview of lipedema. It will cover (i) the clinical presentation of lipedema and (ii) the emerging understanding of the pathophysiological mechanisms that contribute to disease onset and progression and will address (iii) the diagnostic challenges and the lack of standardized diagnostic criteria. The review will also evaluate (iv) current treatment modalities—ranging from conservative approaches such as compression therapy and manual lymphatic drainage to more invasive options like liposuction—and (v) explore whether molecular and metabolic differences between distinct AT depots, particularly abdominal versus femoral fat, parallel the depot‐specific characteristics observed in classical obesity. This comparative approach may yield insights into the unique biology of lipedema fat and help distinguish it from obesity‐related adiposity.

Finally, this review aims to dispel (vi) common misconceptions about lipedema, such as its misclassification as lifestyle‐induced obesity and to provide clear, evidence‐based guidance for both clinicians and patients. By increasing awareness and fostering a deeper understanding of the disease, we hope to advance clinical recognition, promote earlier diagnosis, and ultimately improve outcomes for individuals living with lipedema.

## 2 Methods

An extensive literature search was conducted using multiple electronic databases, including Medline, PubMed, EMBASE, the Cochrane Register of Systematic Reviews, and the Science Citation Index, to identify studies related to the pathophysiology, management, and treatment of lipedema. Tailored search strategies were developed for each database to maximize retrieval of relevant publications from 1940 to 2025. Search terms included combinations of keywords such as “lipedema,” “obesity,” “adipose tissue expansion,” and “body fat distribution.” Additional studies were identified by screening the reference lists of relevant reviews and primary research articles. Only peer‐reviewed articles published in English were considered. The search strategy aimed to capture both foundational studies and recent advances to provide a thorough and current understanding of the topic.

## 3 Clinical Presentation

**FIGURE 1:**
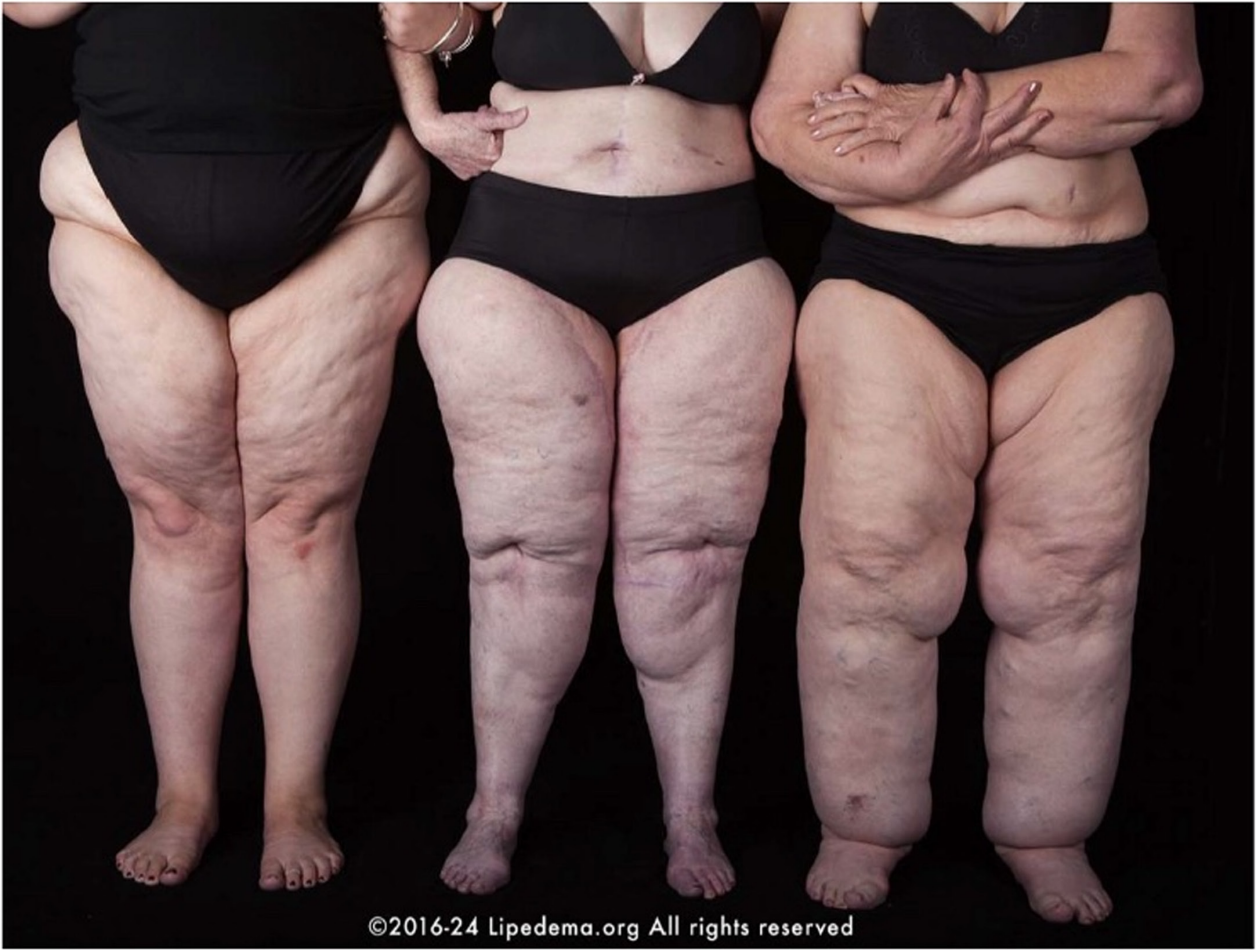
Clinical presentation of lipedema in women. The image is kindly provided by the Lipedema Foundation.

The diagnosis of lipedema is primarily clinical and relies on specific criteria that include (i) bilateral, disproportionate fat deposition primarily in the lower extremities and sometimes the arms while sparing hands and feet; (ii) a granular or nodular texture in the affected areas; (iii) sensitivity to pressure in the affected regions, with patients frequently reporting persistent pain and tenderness; (iv) capillary fragility resulting in bruising even with minimal trauma; and (v) absence of pitting edema [1, 2] (Figure 1). Additionally, although not diagnostic, (i) several tools can support lipedema evaluation such as imaging (e.g., ultrasound, magnetic resonance imaging (MRI), and lymphoscintigraphy) [32, 33, 34], which may show thickened hypodermis without dermal backflow, helping to exclude lymphedema; (ii) body composition analysis can reveal disproportionate limb fat; and (iii) functional assessments, such as quality of life or physical function scores, help assess disease impact and progression.

Lipedema progresses through four clinical stages, each marked by distinct histopathological and immunological features [35]. In Stage 1, the skin appears smooth, and the subcutaneous AT is soft and thickened, with small palpable nodules. Early adipocyte hypertrophy is present, although fibrosis is minimal. Stage 2 is characterized by an uneven skin surface and the presence of larger nodules, accompanied by the onset of interstitial fibrosis and progressive adipocyte enlargement. By Stage 3, pronounced fat deposition leads to large lobules and visible limb deformities. Histologically, there is substantial adipocyte hypertrophy, dense fibrotic matrix accumulation, and increased infiltration of alternatively activated (M2‐like) macrophages. Stage 4, also referred to as lipo‐lymphedema, involves secondary lymphatic dysfunction, chronic edema, and dermal fibrosis.

A hallmark feature of lipedema is the abnormal gynoid distribution of subcutaneous AT, which predominantly affects hips, thighs, and legs and occasionally the arms while typically sparing the hands and feet. This symmetrical fat deposition results in a disproportionate body shape that distinguishes lipedema from other AT disorders, as well as from obesity or lymphedema (Table 1). We recently conducted a comprehensive evaluation of body composition in women with obesity (Obese) (BMI: 30–49.9 kg/m^2^) and obesity and lipedema (Obese‐LIP). Study subjects (Obese vs. Obese‐LIP) were matched on age, BMI, and body fat percentage to assess total body fat mass and fat distribution using multiple imaging methods [21]. Using dual‐energy X‐ray absorptiometry, the study quantified total body fat as well as specific fat depots in the upper and lower body. In Obese‐LIP, there was a pronounced increase in fat mass in the lower extremities, particularly in the thighs and legs, leading to a higher gynoid‐to‐android fat ratio compared to the control group (Obese). MRI provided further insight into fat distribution by measuring abdominal and thigh fat depots. Although Obese‐LIP and controls shared similar abdominal subcutaneous AT (ASAT) and intraabdominal AT (IAAT) volumes, significant differences were observed in thigh subcutaneous AT (TSAT); the Obese‐LIP group had a markedly higher TSAT compared to controls, reinforcing the notion that lipedema is characterized by lower body adiposity. MRI was used to evaluate intrahepatic triglyceride (IHTG) content, which was found to be comparable between groups, suggesting that the increase in subcutaneous AT in lipedema is not exclusively attributable to an increase in lower body fat. The same study also evaluated whether moderate diet‐induced weight loss impacted body fat in lipedema. A relative decrease (12%) in total body fat mass, leg fat mass, TSAT, and ASAT volumes was reported in the Obese‐LIP group. In individuals with classical obesity, intentional weight loss typically leads to about 75% of the weight reduction coming from a decrease in body fat [36], across subcutaneous abdominal and thigh AT depots [37, 38, 39]. AT depots associated with lipedema are thought to be resistant to negative energy balance, which may explain the limited reduction in lower body fat mass observed after weight loss [40, 41]. However, the proportion of total fat mass loss from leg fat (~33%) in the Obese‐LIP group is consistent with the ~30% contribution observed in women with obesity after similar weight loss [42, 43]. In addition, results from this study are in line with a previous study that found minimal (~3%) weight loss resulted in a decrease in leg fat mass in women with obesity and lipedema without any difference in the reduction in leg fat mass compared to controls [44]. These results challenge the prevailing notion that lipedema fat is resistant to negative energy balance and highlight the importance of diet‐induced weight loss in the medical management of individuals with both obesity and lipedema.

**TABLE 1:** Comparison between lipedema, obesity, and lymphedema.

	Lipedema	Obesity	Lymphedema
Definition	A chronic condition characterized by abnormal fat accumulation, usually in the lower extremities, with or without upper extremity involvement	A condition marked by excessive body fat accumulation that exceeds normal levels	A condition involving lymphatic fluid accumulation in tissues, leading to swelling due to impaired lymphatic drainage
Primary affected areas	Primarily the lower limbs but can affect the upper limbs, typically symmetrical	Can affect any area of the body, often in the abdomen, thighs, and hips but can be more generalized	Primarily affects the limbs (legs and arms) but can also involve other areas (genital region and abdomen)
Fat distribution	Symmetrical and disproportionate fat distribution in specific areas, often “column‐like” legs	Generalized fat distribution throughout the body, often centered around the abdomen and thighs	Localized fat accumulation (often in the form of hard, fibrotic tissue) alongside swelling in the affected limbs
Edema (fluid retention)	No significant fluid retention unless lymphatic dysfunction is present	No significant edema, although fluid retention may occur in cases of metabolic dysfunction	Severe, persistent edema due to fluid accumulation in the affected limbs, with no pitting edema in later stages
Pain	Often painful, with tenderness or sensitivity to touch, especially in affected limbs	Generally not painful unless there are associated conditions like joint pain, metabolic disorders, or other complications	Can be painful due to swelling, stiffness, and discomfort, especially in the later stages
Skin changes	The skin may become fibrotic, and “skin dimpling” or “orange peel” appearance may occur in advanced stages.	Skin changes are generally absent unless there are associated complications like cellulitis.	Thickened, hardened skin (fibrosis), often with a *peau d'orange* (orange peel) appearance
Lymphatic function	Normal lymphatic function initially but may be impaired in advanced stages, contributing to fluid retention	Normal lymphatic function unless associated with metabolic complications	Impaired lymphatic function due to blockage or damage of lymphatic vessels, leading to swelling
Cause	Genetic, hormonal, and vascular factors, often worsened by obesity or physical inactivity	Genetic, environmental, and lifestyle factors (diet, lack of exercise, etc.)	Often secondary to cancer treatments, infections, or trauma that damages lymphatic vessels; primary lymphedema may be genetic.
Management/treatment	Weight management, compression therapy, structured exercise, lymphatic drainage, and liposuction in advanced cases	Weight loss through diet, exercise, and behavioral modifications; bariatric surgery in some cases	Manual lymphatic drainage (MLD), compression garments, elevation, and surgical options (e.g., lymphatic bypass surgery or liposuction in some cases)
Progression	Slowly progressive; may worsen with weight gain, hormonal changes, or aging	Progressive if weight is not controlled but may stabilize or improve with lifestyle changes	Progressive if untreated, with increased swelling, skin fibrosis, and potential disability

Women with lipedema have been anecdotally reported to have a lower risk of developing metabolic syndrome, prompting a recent study to evaluate their metabolic function in comparison to women with obesity and to women who are lean. Key metabolic parameters included plasma lipid profiles to evaluate dyslipidemia, an oral glucose tolerance test (OGTT) to assess glucose metabolism, and a hyperinsulinemic–euglycemic clamp procedure to measure whole‐body insulin sensitivity. The three different groups were defined as (i) the Lean group: BMI 18.5–24.9 kg/m^2^, fasting plasma glucose concentration < 100 mg/dL, 2‐h OGTT plasma glucose concentration < 140 mg/dL, and HbA1c ≤ 5.6% (≤ 38 mmol/mol); (ii) the Obese group: BMI 30–49.9 kg/m^2^, fasting plasma glucose concentration < 126 mg/dL, 2‐h OGTT plasma glucose concentration < 200 mg/dL, and no diagnosis or features of lipedema; and (iii) the Obese‐LIP group: diagnosis of lipedema based on the criteria of Wold et al. [40], BMI 30–49.9 kg/m^2^, fasting plasma glucose concentration < 126 mg/dL, and 2‐h OGTT plasma glucose concentration < 200 mg/dL. The Obese and Obese‐LIP groups were matched on age, BMI, total body fat mass, and percent body mass as fat. The study found no significant differences between the Obese and Obese‐LIP groups in fasting plasma lipid profiles, glucose, insulin, and C‐peptide concentrations, as well as in 2‐h post‐OGTT plasma glucose levels, HbA1c, or hepatic insulin sensitivity. However, whole‐body insulin sensitivity was approximately 48% greater (*p* < 0.05) in the Obese‐LIP group compared to the Obese group, suggesting a relative preservation of insulin sensitivity in women with lipedema. When compared with the Lean group, the Obese‐LIP group exhibited markers of metabolic dysfunction, including higher fasting plasma glucose, C‐peptide, triglyceride, and 2‐h OGTT glucose concentrations, elevated HbA1c, lower HDL cholesterol levels, and reduced hepatic and whole‐body insulin sensitivity. These findings suggest that although women with lipedema may have better insulin sensitivity than BMI‐matched women with obesity, they still exhibit significant metabolic impairments when compared to metabolically healthy lean individuals. This study provides critical insights into the metabolic profile of lipedema, reinforcing the need for further research to understand its implications for disease progression and potential therapeutic interventions.

Lipedema subcutaneous AT exhibits several unique characteristics that distinguish it from other conditions characterized by fat expansion, such as obesity or lymphedema [2]. These features, which highlight its distinct pathophysiology and clinical presentation, include (i) *nonpitting nature*: Unlike lymphedema, where swelling often creates pitting when pressed, lipedema subcutaneous AT remains firm and nonpitting, reflecting lack of significant interstitial fluid accumulation; (ii) *anatomical distribution*: The abnormal adiposity characteristically stops at the ankle, creating a distinct cuff‐like appearance at the malleoli that highlights the condition's localized effect on the lower extremities, sparing the feet, which is not typically seen in generalized obesity; (iii) *granular texture*: Palpation reveals a “sand grain” feel, possibly due to the presence of micronodule or macronodules within the AT; (iv) *nodular phenotype*: In more advanced cases, the subcutaneous AT may feel like “beans in a bag,” reflecting larger nodules or fibrotic changes in the tissue; and (v) *easy bruising*: Patients with lipedema are prone to easy bruising, indicative of fragile subdermal capillaries [2]. This vascular fragility may stem from microvascular dysfunction, such as weakened capillary walls, increased permeability, or altered extracellular matrix (ECM) support. Telangiectasias or spider veins in lipedema‐affected areas further point to capillary fragility and localized vascular abnormalities. These features are in line with the proposed hypotheses of compromised microvascular integrity contributing to lipedema pathophysiology.

Pain or a sensation of heaviness in the affected extremities is a defining feature of lipedema, clearly distinguishing it from conditions like obesity or lymphedema, which are typically nonpainful. Although the exact etiology of lipedema pain remains unclear, it is believed to result from a combination of chronic inflammation, mechanical stress, and possibly neuropathic alterations [45, 46]. A recent study by Dinnendahl et al. [47] assessed psychometric and/or sensory alterations in nonobese women with lipedema and in BMI‐matched controls according to the protocol of the German Research Network on Neuropathic Pain [48]. The study found that all participants scored within normal ranges for depression, anxiety, and stress (measured with the DASS questionnaire), suggesting that stress did not significantly influence their pain experiences. However, lipedema patients reported a significantly lower quality of life in terms of social, mental, and physical functioning, consistent with previous findings [49]. Additional results from the study revealed altered sensory thresholds, further differentiating lipedema from obesity and lymphedema. Specifically, the *z*‐score for pressure pain threshold (PPT), measured at the quadriceps femoris and thenar eminence, was reduced by twofold, aligning with the reported tenderness and discomfort in the affected extremities. The *z*‐score for vibration detection threshold (VDT), assessed at the patella and the processus styloideus ulnae, was increased by two and a half times, indicating altered somatosensory processing and sensory nerve dysfunction. This change may contribute to the characteristic sensations of heaviness and discomfort observed in women with lipedema. Notably, both thresholds were selectively altered in the affected thigh but not in the unaffected hand. Pressure pain is hypothesized to be mediated by small or medium diameter C/Aδ fibers, whereas vibration is mediated by large diameter Aβ fibers [50]. The absence of abnormalities in quantitative sensory testing in women with lipedema implies that sensory nerve conduction, stimulus detection, and transmission were intact. Because central integration appeared unaffected in women with lipedema, it is plausible that systemic sensitization (e.g., from systemic inflammation or hormonal changes) may not play a primary role in lipedema pain. It has been proposed that a more rigid ECM might amplify pressure transmission, leading to increased nociceptive fiber activation, despite the paradoxical expectation that tissue amassing would dampen pressure transmission [47]. An increased collagen density or altered ECM composition, as recently reported [21], could create a stiffer microenvironment, enhancing pressure transmission to nociceptive fibers (C/Aδ). ECM rigidity may also impair lymphatic function, leading to localized edema and subtle tissue ischemia, which might indirectly contribute to pain. Although the exact cause of lipedema pain remains speculative, these findings narrow the scope of possible mechanisms. The focus on ECM rigidity and mechanotransduction pathways offers promising avenues for future investigation.

## 4 Lipedema Pathogenesis and Pathophysiology

Lipedema was first described in the 1940s [40, 51]; however, disease pathogenesis and pathophysiology remain poorly understood continuing to pose challenges for diagnosis, management, and treatment development. Several studies have now consistently reported that AT expansion in lipedema is associated with adipocyte hypertrophy [31, 52]. Adipose‐derived stem cells (ADSCs) isolated from lipedema AT and grown in a 2D monolayer exhibit a higher adipogenic differentiation potential compared to ASCs isolated from healthy individuals [53]. When comparing lipedema and non‐lipedema ADSCs, transcriptional profiling revealed differential expression of over 3400 genes, many of which are involved in ECM and cell cycle/proliferation signaling. Of note, Bub1, also known as Benzimidazole 1, was found to be upregulated in lipedema ADSCs. Bub1 encodes a cell cycle regulator that is central to the kinetochore complex and controls various histone proteins involved in cell proliferation [54]. Downstream signaling analysis of lipedema ADSCs showed enhanced activation of histone H2A, a critical driver of cell proliferation and a target of Bub1. Notably, hyperproliferation in lipedema ADSCs was suppressed by the serine/threonine kinase inhibitor 2OH‐BNPP1, which is a Bub1 inhibitor, and following CRISPR/Cas9‐mediated depletion of the Bub1 gene [55]. A different study, using an elegant and comprehensive multiomics approach, reported a localized reduction in inflammatory signaling coupled with enhanced mitochondrial function in lipedema AT [56]. In addition, metabolomic and lipidomic profiling showed broader metabolic disturbances, including altered levels of glutamic acid, glutathione, and sphingolipids [56].

Lower body fat accumulation (gluteofemoral obesity) is usually associated with protective effects against cardiovascular disease and type 2 diabetes mellitus when adjusted for total fat mass [57]. Conversely, upper body fat accumulation, commonly referred to as abdominal obesity, is strongly linked to an elevated risk of cardiovascular disease [58], insulin resistance [59], type 2 diabetes mellitus [60], and even all‐cause mortality [61]. Lower body fat exhibits slower lipid turnover, an enhanced capacity to accommodate redistributed fat in response to weight gain [62]. Abdominal fat depots, instead, are marked by rapid uptake of diet‐derived lipids and high lipid turnover [63, 64], which is readily stimulated by adrenergic receptor activation [65]. Lower body adiposity also demonstrates fewer signs of inflammatory insult, reinforcing its protective role in metabolic health [57, 66, 67]. Pinnick et al. [66] have elegantly shown that functional differences between upper and lower body adipose depots are regulated by site‐specific developmental genes including members of the homeobox (HOX) family (e.g., SHOX2 and IRX2), and T‐box genes (e.g., TBX15 and TBX5) [66]. These transcriptional regulators are generally involved in early embryonic development, body patterning, and cell specification. The HOX genes may regulate differentiation of adipocytes [68]. *HOXA6*, *HOXA5*, *HOXA3*, *IRX2*, and *TBX5* are highly expressed in subcutaneous abdominal AT, whereas *HOTAIR*, *SHOX2*, and *HOXC11* are instead highly expressed in the gluteofemoral AT [66]. These genes are regulated through epigenetic mechanisms however, the meaning of these regional signatures of developmental genes remains unclear, and whether they actively influence functional characteristics of AT remains to be determined in lipedema fat.

**FIGURE 2:**
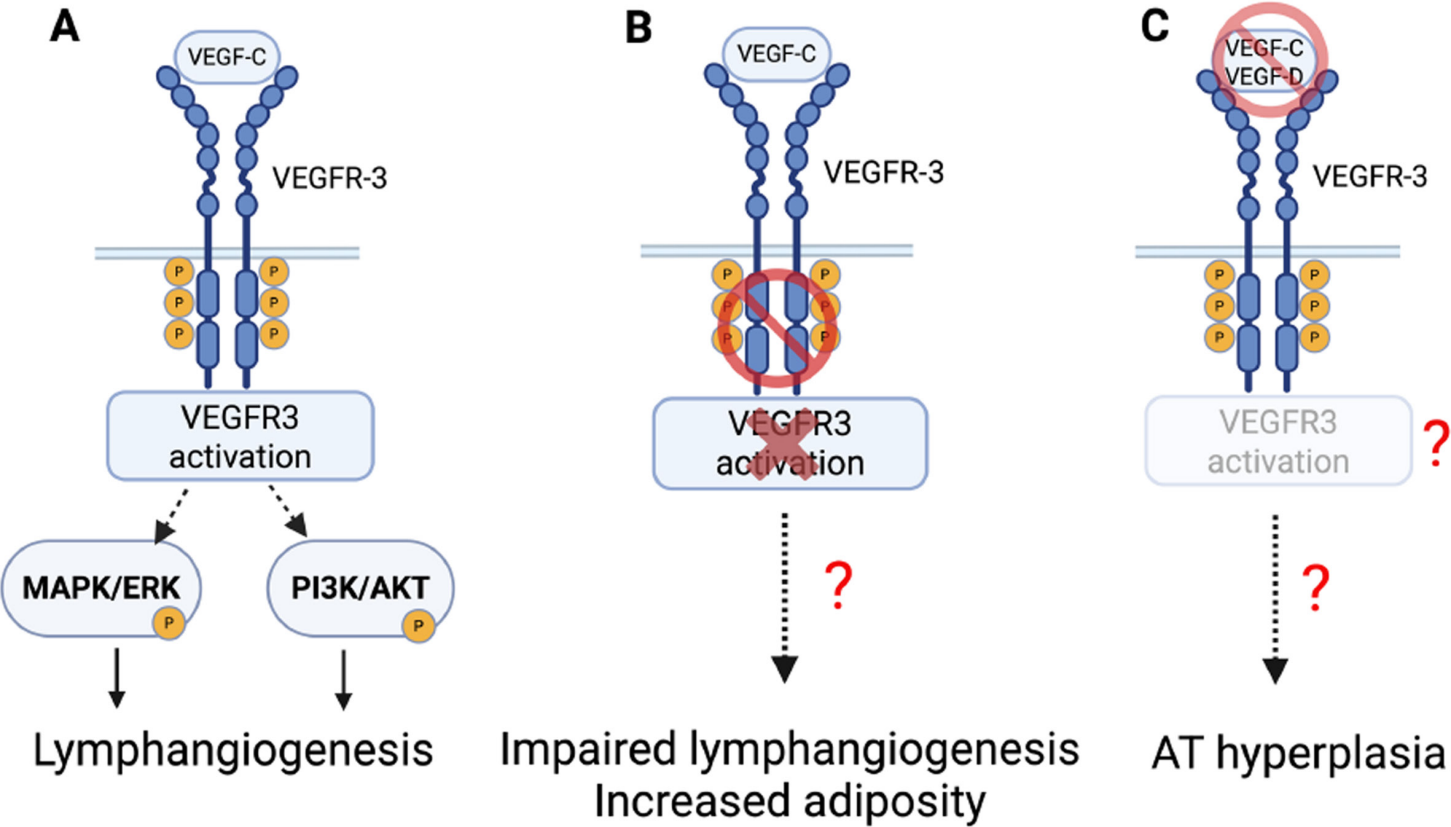
VEGF‐C/VEGFR3 signaling pathway regulates adiposity. (A) VEGF‐C binds to VEGFR3 activating MAPK/ERK and PI3K/AKT signaling pathways important for lymphangiogenesis. Altered VEGF‐C/VEGFR3 axis due to either (B) VEGFR3 tyrosine kinase inactivation or (C) blockade of VEGF‐C (or VEGF‐D) affects AT remodeling in preclinical mouse models.

Our recent study investigated whether the molecular and metabolic differences between abdominal and femoral AT depots in lipedema resemble those observed in classical obesity [21]. Bulk RNA sequencing compared TSAT and ASAT in women with obesity and lipedema (Obese‐LIP). The study found that TSAT exhibits a downregulation in biological pathways related to developmental programming and specification that included genes such as *HOXA3*, *HOXA5*, and *IRX2* and in pathways related to lymph/angiogenesis. In contrast, TSAT showed an upregulation of pathways related to immune response and inflammation. These findings suggest that although TSAT in lipedema may be inflammatory in nature, its metabolic profile and pathophysiological mechanisms could differ significantly from fat expansion in classical obesity. Specifically, lipedema‐related fat expansion may be driven more by structural and functional changes of the lymphatic and vascular systems rather than the metabolic dysfunction typically observed in classical obesity. Lipedema TSAT showed reduced expression of genes (i) *VEGFC*, vascular endothelial growth factor (VEGF)–C, and its cognate receptor *FLT4* (protein is VEGFR‐3), which have canonical roles in blood and lymphatic vessel proliferation and function [69, 70, 71]; (ii) *CLD11*, claudin 11, which regulates lymphatic valve development [72]; (iii) *SOX17*, which is important for endothelial regeneration following injury [73, 74]; (iv) *THBS4*, thrombospondin 4, which regulates endothelial ECM interaction [75]; and (vi) *ANG*, angiogenin, which regulates angiogenesis [76]. AT expansion in preclinical models of obesity is associated with lymphatic vascular disorganization, as well as irregular lymphatic branching and rarefication [77, 78, 79], resulting in impaired lymphatic drainage, interstitial fluid stasis, and immune cell trapping. Conversely, deletion of key lymphangiogenic genes in preclinical models is often associated with abnormal AT expansion and fat accumulation in tissues. VEGFR3 is a tyrosine kinase receptor encoded by the *FLT4* gene that regulates lymphangiogenesis [80]. VEGF‐C binding to VEGFR3 induces receptor dimerization, autophosphorylation, and internalization and activates PI3K/AKT and MAPK/ERK signaling pathways important for lymphangiogenesis [81] (Figure 2a). Our group and others have shown that aberrant VEGFR3 signaling in preclinical models due to a mutation inactivating the receptor tyrosine kinase is associated with leaky lymphatics and fat accumulation in tissues [82, 83] (Figure 2b). In a different study, the blockade of circulating VEGF‐C and VEGF‐D in mice was associated with metabolically healthy AT expansion during a high‐fat diet [84], mimicking key clinical features of lipedema (Figure 2c). Elevated levels of circulating VEGF‐C^85^ are reported in lipedema compared to age‐ and BMI‐matched controls, but it is unclear whether VEGFR3 signaling remains functional. The combination of downregulated lymphangiogenic/angiogenic genes in the Obese‐LIP cohort is in line with the clinical features of interstitial fluid accumulation [86], along with increased tissue sodium and adipose deposition [87, 88, 89] commonly observed in lipedema, suggesting that compromised lymphatic function plays a central role in the disease's pathophysiology. This dysfunction likely leads to a cycle of fluid retention and localized swelling, which worsens as lymphatic bulk transport becomes increasingly impaired [90, 91, 92, 93]. However, reports of lymphatic anatomy in lipedema are inconsistent, ranging from normal [85] to an increase in lymphatic vessel area [31] or dilated lymphatics [34] to lymphatic microaneurysms [94]. These discrepancies could be related to differences in the methods used to assess lymphatic anatomy and function, inadequate numbers of participants to detect statistically significant effects, and inconsistent criteria to match patients and controls.

Alterations in the function of both vascular and lymphatic vessels play a crucial role in the accumulation of immune cells and inflammatory cytokines within affected tissues. These changes disrupt normal fluid balance and promote a pro‐inflammatory environment, which in turn exacerbates tissue inflammation [77]. Ultrastructural analysis by electron microscopy has revealed endothelial abnormalities that may underlie adipocyte hypertrophy, disrupted calcium metabolism, and macrophage infiltration [95]. These findings suggest that endothelial dysfunction, marked by increased endothelial proliferation and pericyte density, contributes to the pathological remodeling of lipedema‐associated AT [31, 96]. It has been hypothesized that the continuous accumulation of immune cells, coupled with sustained cytokine release, perpetuates a cycle of localized inflammation, potentially contributing to tissue damage and the progression of lipedema [1]. In line with this, it was recently reported that the Obese‐LIP cohort exhibits increased pro‐inflammatory M1‐like macrophage infiltration in TSAT assessed by flow cytometric analysis and increased expression of genes involved in inflammation, including macrophage and T cell markers, as well as markers of T cell activation compared to ASAT [21]. However, these findings are in conflict with Wolf et al [97] reporting a shift in AT macrophages toward a more immunosuppressive M2‐like state characterized by high levels of immunosuppressive markers CD206, CD163, and Clever‐1. The differences in results could be due to differences in experimental design and methodology to profile macrophages and stage of lipedema.

A compelling hypothesis for the pathogenesis of lipedema suggests that improper remodeling of the ECM could play a central role in abnormal AT expansion. The composite expression of genes encoding 12 collagen isoforms and expression of key regulators of AT fibrosis and fibrogenesis—such as *LOX1*, *SEMA3C*, *CCN2*, and *VCAN*—were all greater in TSAT than ASAT [21]. These findings are consistent with prior reports of a fibrotic profile in lipedema fat [35, 52]. In particular, the uncoupling of the matrix metallopeptidase 14, MMP‐14‐caveolin 1 axis in adipocytes may disrupt normal matrix processing, leading to aberrant ECM remodeling [98]. This disruption could drive the hypertrophic expansion of subcutaneous AT, contributing to the abnormal fat deposition characteristic of lipedema. Women with lipedema often report symptoms such as joint hypermobility [99], reduced skin elasticity [100], and aortic stiffness [101], all of which suggest a potential involvement of connective tissue dysfunction. A reduction in the expression of the *ELASTIN* gene was found in lipedema TSAT compared to ASAT [21]. Interestingly, individuals with Williams syndrome, a connective tissue disease where a deletion of the elastin gene has been implicated [102], develop a lipedema‐like phenotype in the lower extremities. These observations point to possible alterations in the structural properties of connective tissues, which may contribute to the clinical features of lipedema.

Lipedema increases the risk of developing VTE [5] compared to BMI‐matched control, supporting the hypothesis that elevated BMI alone may not be the primary driver of thrombosis [103]. Recent analyses of platelet transcriptomes from patients with lipedema as well as from those with lymphedema have revealed important differences in gene expression that may offer valuable insights into the distinct pathophysiological mechanisms underlying each condition. The transcriptomic profile of platelets from lipedema patients showed a greater expression of genes related to glycolipid modifications, protein dephosphorylation, and cytoplasmic protein translation. In contrast, platelets from patients with lymphedema displayed reduced signals for angiogenesis and inhibited mitosis [6]. Elevated levels of platelet factor 4 (PF4/CXCL4) were reported in circulating blood plasma exosomes of patients with lipedema [104]. PF4 is a chemokine released by activated platelets, involved in coagulation, inflammation, and vascular remodeling [105, 106]. Elevated PF4 levels in blood plasma exosomes strongly correlate with lymphatic dysfunction, making it a reliable indicator of lymphatic pathology. This finding not only supports the hypothesis that lymphatic defects might be an important contributor to lipedema pathophysiology but also provides a potential diagnostic tool.

## 5 Treatment Options for Lipedema

In the absence of an etiological treatment, a multidisciplinary approach is essential to optimize outcomes for patients with lipedema. Current strategies focus on alleviating symptoms, improving function, and preventing disease progression rather than addressing the root cause of the condition. Collaboration between healthcare providers, including specialists in vascular medicine, physical therapy, psychology, and surgery, is crucial to address the complex needs of these patients [7, 107].

### 5.1 Compression Therapy and Complex Decongestive Lymphatic Therapy

Compression therapy and complex decongestive lymphatic therapy (CDP) represent cornerstone interventions in improving symptoms and enhancing the quality of life. Compression garments help alleviate the tenderness and heaviness of affected limbs by improving tissue support, venous return, and lymphatic drainage, which overall reduce limb swelling. CDP instead is particularly effective for patients with lipo‐lymphedema—a common complication of lipedema involving concurrent lymphatic dysfunction. CDP consists of (i) manual lymph drainage (MLD), a specialized massage technique to stimulate lymphatic flow and reduce swelling; (ii) multilayered and multicomponent compression bandaging, essential for maintaining postdrainage volume reduction and improving venous return; and (iii) skincare, which prevents skin breakdown and infections that may exacerbate symptoms. Intermittent pneumatic compression can enhance the effects of CDP by improving venous flow and decreasing lymph production [108]. A clinical study comparing CDP with and without IPC demonstrated significant lower limb volume reduction in both groups, with reductions of 6.2% and 8.9%, respectively, highlighting the utility of IPC as a complementary therapy, particularly for patients requiring additional volume reduction.

### 5.2 Structured Exercise

Although fat volume remains unaffected, strength training and low‐impact activities are reported to alleviate pain and improve mobility [109, 110]. A consensus statement from the Italian Society of Motor and Sports Sciences (SISMeS) and the Italian Society of Phlebology (SIF) emphasizes the importance of structured exercise as a key component in the conservative management of lipedema [111]. According to this consensus, personalized exercise programs that integrate muscle strengthening and flexibility training are essential for addressing the multifaceted challenges of lipedema. These programs should be tailored to each individual's disease stage and physical capacity, ensuring a gradual and safe progression to optimize therapeutic outcomes.

Structured exercise training, particularly water‐based exercise, has been shown to offer both physiological and psychological benefits for individuals with lipedema because of its low‐impact nature, which reduces joint stress while promoting lymphatic drainage, enhancing functional mobility, and providing joint relief. These effects are crucial in managing lipedema symptoms, which often include pain, limited mobility, and fluid retention. Additionally, such exercise aids in weight control, muscle strengthening, and improving flexibility. Beyond its physical benefits, structured exercise also contributes significantly to mental well‐being. Research has demonstrated improvements in self‐esteem, mood, and overall quality of life, which are especially important for individuals with lipedema who may face emotional challenges related to the visible effects of the condition [112]. Incorporating exercise into a comprehensive care plan can address both the physical and psychological aspects of the disease, promoting long‐term adherence to treatment and improving overall patient self‐management [113].

Despite these promising interventions, there is a critical need for large‐scale studies to establish standardized exercise prescriptions tailored to the various stages of lipedema. Such research would enhance our understanding of optimal exercise strategies, ensuring that treatment plans are evidence‐based, individualized, and effective in improving the quality of life for individuals affected by this often misunderstood condition.

### 5.3 Nutritional Approaches

It is widely believed that the AT depots affected by lipedema are resistant to negative energy balance, making it difficult to reduce lower body fat mass through weight loss. However, recent findings challenge this assumption and provide new insights into the role of weight management in lipedema [44, 114, 115, 116]. Positive effects on lipedema body composition and diet‐induced weight loss have been reported in one case study [117] and interventional studies [44, 116] using a ketogenic, low‐carbohydrate diet or a Mediterranean diet. A recent study found that diet‐induced weight loss induces a similar (~12%) relative decrease in total body fat mass, leg fat mass, TSAT volume, and ASAT volume in women with obesity and lipedema [21] and that the proportion of total fat mass loss from leg fat (~33%) in the Obese‐LIP group is consistent with the ~30% contribution previously observed in women with obesity after similar weight loss [42, 43]. These results align with earlier studies showing that even minimal weight loss (~3%) in women with obesity and lipedema resulted in a significant reduction in leg fat mass, comparable to women without lipedema [44]. Therefore, these results contradict the dogma that lipedema fat is resistant to negative energy balance supporting the importance of diet‐induced weight loss in the medical management of people with obesity and lipedema.

Women with lipedema exhibit relatively preserved insulin sensitivity compared to BMI‐matched women with obesity [21]. Furthermore, moderate diet‐induced weight loss (~9%) in the Obese‐LIP group led to significant metabolic improvements, including increased hepatic and whole‐body insulin sensitivity, alongside reductions in IHTG content and plasma LDL cholesterol concentration. These beneficial changes are key factors in reducing the risk of type 2 diabetes and cardiovascular disease. Collectively, these results provide a mechanistic explanation for the lower incidence of type 2 diabetes observed in women with lipedema compared to those with obesity alone [118, 119]. Additionally, the study highlights the therapeutic metabolic benefits of moderate weight loss in women with lipedema, emphasizing its role in improving insulin sensitivity and mitigating cardiometabolic risk factors.

Diet‐induced weight loss (~9%) did not significantly alter the expression of genes associated with inflammation or ECM remodeling and lymphatics in either ASAT or TSAT [21]. Notably, weight loss was linked to the upregulation of genes involved in angiogenesis and vascular remodeling specifically in TSAT. The observed increase in angiogenic and vascular remodeling in TSAT post‐weight loss—without corresponding changes in lymphatic gene expression—may represent an adaptive mechanism to support tissue repair or restructuring. This warrants further exploration into its functional significance in the context of lipedema and its potential impact on therapeutic strategies. Diet‐induced weight loss in women with lipedema has been reported to improve pain and enhance quality of life [114, 115, 116]. Although the underlying mechanisms remain poorly understood, excess body weight likely contributes to joint stress, heightened inflammation, and increased mechanical strain, all of which exacerbate discomfort and hinder mobility. Therefore, weight management should be emphasized as a cornerstone of a multidisciplinary approach to lipedema treatment to enhance overall well‐being and mitigate disease progression.

### 5.4 Surgical Options

Liposuction is the primary surgical intervention for managing lipedema, particularly in advanced cases where conservative measures are insufficient. The two most employed methods—tumescent anesthesia (TA) liposuction and water‐assisted liposuction (WAL)—are tailored to address the unique characteristics of lipedema fat while minimizing complications [120]. In TA, a solution containing saline, local anesthetics (e.g., lidocaine), and epinephrine is injected into the subcutaneous tissue causing swelling of fat cells for easier removal and constriction of blood vessels, which reduces bleeding. Fat is then gently suctioned from the targeted areas using blunt microcannulas. TA presents many advantages including minimal blood loss due to vasoconstriction from the tumescent solution and enhances precision and safety, particularly in delicate areas. WAL consists of a high‐pressure controlled spray of tumescent fluid that dislodges fat cells from surrounding connective tissues. In WAL, fat and tumescent fluid are aspirated simultaneously. The water jet gently separates fat from tissues, preserving lymphatic structures and reducing postoperative swelling, ideal for areas with dense fibrous fat or those prone to damage during traditional methods. Unlike traditional liposuction, both TA and WAL liposuction use local anesthetics, avoiding the risks associated with general anesthesia, and focus on lymphatic sparing techniques, crucial for lipedema patients to prevent secondary lymphedema. Both methods have their merits, and the choice often depends on the surgeon's expertise, the patient's condition, and the goals of treatment. Long‐term management postsurgery typically includes compression therapy and lifestyle adjustments to maintain results and prevent progression [120].

## 6 Conclusions: Raising Awareness About Lipedema

Lipedema is a chronic, progressive disease that affects millions of individuals worldwide, predominantly women, yet it remains widely underdiagnosed and misunderstood. Characterized by the disproportionate accumulation of subcutaneous fat in the lower and sometimes upper extremities, lipedema is often mistaken for general obesity or lymphedema, leading to misdiagnosis and delayed interventions. The lack of awareness among healthcare providers and the general public results in significant delays in receiving appropriate care, exacerbating symptoms such as pain, osteoarthritis, and mobility limitations. Without timely recognition and management, lipedema can severely compromise an individual's physical health, emotional well‐being, and overall quality of life. A concerted effort is urgently needed to improve awareness and education at multiple levels—among the public, healthcare professionals, and policymakers—regarding the distinguishing features of lipedema, its progression, and its impact on physical and mental health. Public health initiatives and advocacy campaigns should be launched to dispel common misconceptions and highlight the differences between lipedema, obesity, and lymphedema. Additionally, comprehensive research efforts must be prioritized to better understand the underlying pathophysiology of lipedema, which remains poorly characterized. Identifying key molecular and genetic factors contributing to disease onset and progression will be essential in developing targeted therapies. The establishment of standardized diagnostic criteria and the identification of reliable biomarkers are critical steps toward ensuring early and accurate diagnosis. Current diagnostic methods rely heavily on clinical examination, which can be highly subjective and inconsistent across practitioners. Developing evidence‐based guidelines and integrating imaging techniques, molecular profiling, and metabolic assessments will improve diagnostic accuracy and enable personalized treatment strategies.

In addition to advancing research and diagnostics, clinician training programs should be implemented to enhance the ability of healthcare providers to differentiate lipedema from other conditions such as obesity, lymphedema, and metabolic disorders. Equipping physicians, endocrinologists, dermatologists, vascular specialists, and other healthcare professionals with the knowledge and tools to recognize lipedema will lead to earlier interventions and improved patient outcomes.

Multidisciplinary care must also be prioritized to provide comprehensive management strategies for individuals with lipedema. This includes conservative therapies such as compression therapy, manual lymphatic drainage, and exercise programs tailored to lipedema patients, as well as surgical interventions like liposuction for advanced cases. Additionally, addressing the psychological burden of lipedema through mental health support, patient education, and community‐based resources will be essential in improving quality of life. Developing specialized training programs and establishing standardized treatment guidelines will ultimately ensure that individuals affected by lipedema receive the appropriate care and support.

## Author Contributions

Vincenza Cifarelli conceived and wrote the article.

## Conflicts of Interest

V.C. serves on the Scientific Advisory Board for HAB Nutritional Center. No other potential conflicts of interest relevant to this article were reported.
